# Comparative transcriptomic and phenotypic analysis of monoclonal and polyclonal *Populus deltoides* genotypes

**DOI:** 10.3389/fpls.2024.1498535

**Published:** 2025-01-23

**Authors:** Macy Gosselaar, Mark A. Arick, Chuan-Yu Hsu, Heidi Renninger, Courtney M. Siegert, Waqar Shafqat, Daniel G. Peterson, Austin Himes

**Affiliations:** ^1^ Department of Forestry, Forest and Wildlife Research Center, Mississippi State University, Mississippi State, MS, United States; ^2^ Institute for Genomics, Biocomputing, and Biotechnology, Mississippi State University, Mississippi State, MS, United States

**Keywords:** eastern cottonwood, niche differentiation, differential gene expression, nitrogenuse efficiency, productivity

## Abstract

*Populus* species are highly valued for bioenergy and bioproducts due to their rapid growth and productivity. Polyclonal plantings, or mixtures of *Populus* clones, have shown the potential to enhance resource utilization and productivity, likely due to phenotypic differences arising from niche differentiation. In this study, we investigated gene expression and productivity in monoclonal and polyclonal stands of *P. deltoides*. Phenotypic results showed that polyclonal plots exhibited higher leaf area index (LAI; p < 0.01, 2.96 ± 0.057 m^2^) and total biomass (p < 0.01, 2.74 ± 0.06) compared to monoclonal plots, indicating superior productivity. RNA sequencing revealed upregulation of key genes such as exocyst subunit *exo70 family protein H7 (EXO70H7)*, *NDH-dependent cyclic electron flow 5 (NDF5)*, and *expansin-like A3* (*EXLA3*). We also observed enrichment in phenylalanine metabolism and other secondary metabolic pathways in clone S7C8. Phenotypic results, upregulated genes and enriched biological pathways identified in this study may explain the enhanced productivity, increased nitrate content, and expanded canopy in polyclonal plantings. Overall, this study provides a foundation for future research to enhance forest productivity by linking molecular mechanisms to practical applications in field plantings.

## 1 Introduction

*Populus* is an ideal model woody genus for genomic studies of trees (Bradshaw et al., 2000; Taylor, 2002; Tuskan et al., 2006; Jansson and Douglas, 2007). As a model woody plant, *Populus* is a favorable system for understanding diverse physiological and morphological processes, including environmental responses to abiotic and biotic stresses (Popko et al., 2010). The ability of *Populus* to adapt to diverse conditions as well as prominent genetic variations, such as single nucleotide polymorphisms (SNP), has provided researchers with a rich source of variation in *Populus* morphology and physiology (Bradshaw et al., 2000). *Populus* spp. are characterized by rapid juvenile growth, high productivity, and significant water requirements (Hamanishi et al., 2015). Further, their short-term physiological responses to environmental variables are rapid and pronounced, producing distinctive tree phenotypes in 1-3 years in field environments (Bradshaw et al., 2000; Brunner et al., 2004). Another attribute of the *Populus* genomic system is its ecological and interspecific diversity. Levels of genetic diversity are high for molecular markers, such as simple sequence repeats for marker assisted selection, and for adaptive traits including vegetative phenology (Brunner et al., 2004). For example, assessing tissues, like leaves, at the genomic level may provide information on vegetative productivity and differentiation of photosynthetic activity between clonal varieties.

Additionally, *Populus* spp. help solve a number of large-scale problems, e.g., through the mitigation of pollution runoff, providing a source of carbon neutral energy, and supporting the green economy (Forrester et al., 2005; Richards et al., 2010). Phytoremediation using *Populus* to clean up contaminated groundwater is a common phytotechnology, and these trees are effective at controlling the migration of pollutants, including in riparian areas (Zalesny et al., 2012). Compared to herbaceous plants, *Populus* spp. are excellent candidates for remediation as they quickly produce high biomass in stems and leaves, allowing them to store large amounts of pollutants, including agricultural nitrogen runoff (Shim et al., 2013). Similarly, planting *Populus* spp. in riparian zones, which are known to function as buffers, could reduce other non-point source pollution from agricultural lands to streams, improving water quality (Hefting et al., 2005, 2006).

Identifying *Populus* genotypes that demonstrate both productivity and resilience to climate change is crucial for ensuring forest sustainability, as trees worldwide face increasing environmental challenges (Niemczyk et al., 2019). Transcriptome analysis plays a key role in deciphering *Populus* genetic networks and establishing molecular biomarkers that respond to increased productivity and environmental challenges, including pollution and abiotic stress (Jiang et al., 2015). Phenotypic plasticity of organisms like *Populus* spp., including physiological and developmental plasticity, may arise from alternative transcription initiation, including co-transcriptional regulatory mechanisms, which modify gene expression levels (de Klerk and ‘t Hoen, 2015). Functional genomics can reveal direct changes in the expression of individual genes, enhancing the ability to connect specific genes to various phenotypes observed in field trials of *Populus* spp (Brunner et al., 2004). Genomic studies can be conducted in greenhouse conditions but tend to have more limited populations, tree sizes, and physiological characteristics (Brunner et al., 2004). On the other hand, field trials allow researchers to evaluate productivity and identify potential ecosystem impacts of *Populus* spp. (Zalesny et al., 2012). Furthermore, field-based studies account for the inherent complexity of gene-environment interactions, including genotype-specific responses to factors such as nutrient availability, and biotic interactions (Richards et al., 2010). These interactions contribute to differential gene expression patterns that are difficult to replicate in controlled greenhouse settings. While transcriptomic field studies can be challenging, they provide an unparalleled opportunity to uncover and confirm complex gene expression responses to diverse environmental conditions (Izawa, 2015; Kuchma et al., 2020). Such studies can help identify context-dependent genetic mechanisms that drive resilience and productivity, which are essential for translating genomic discoveries into real-world applications for forest sustainability and climate adaptation strategies.

**Figure 1:**
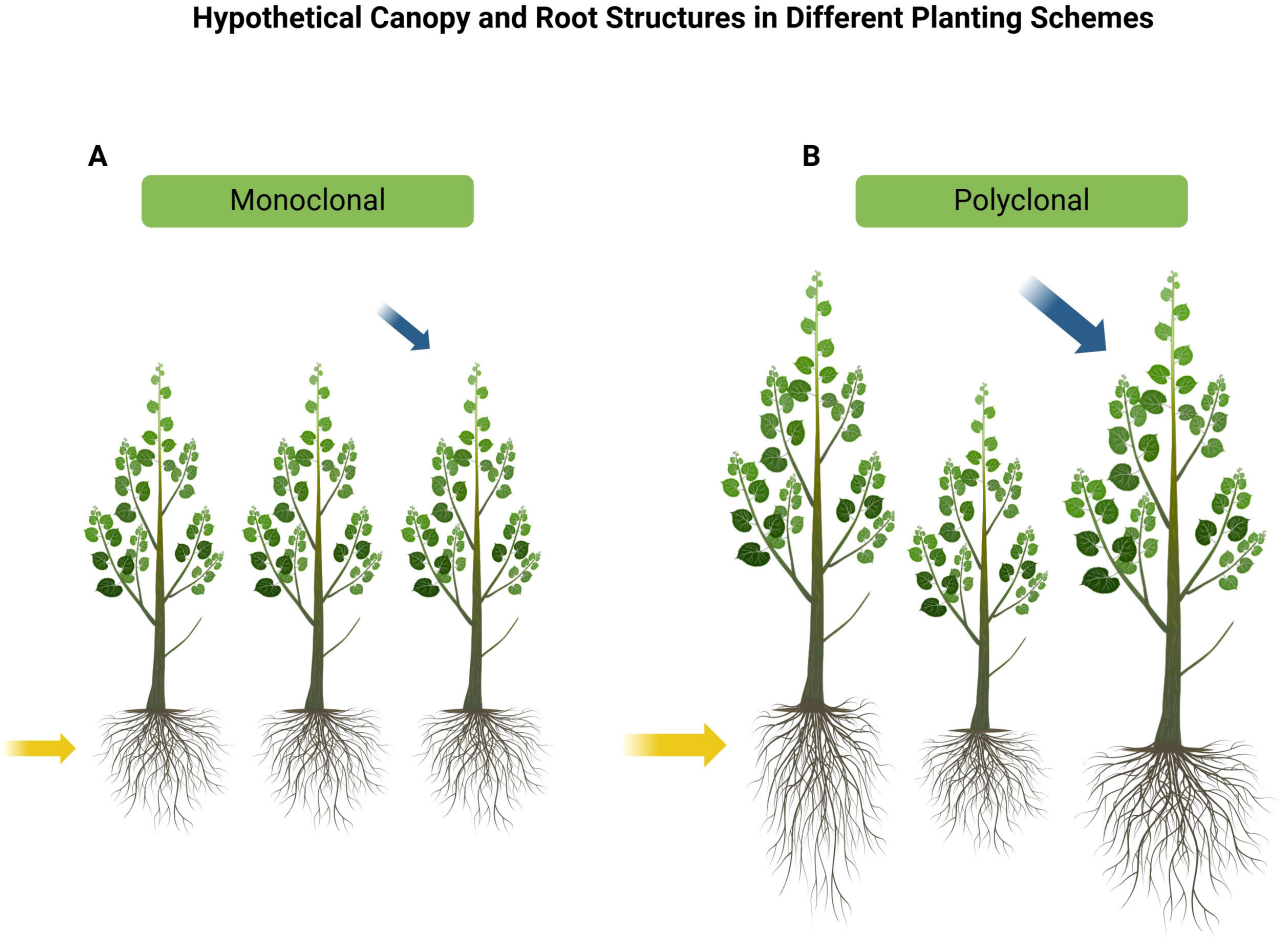
**(A)** Monoclonal and **(B)** polyclonal plantings and their hypothetical canopy and root structures. Morphological differences, such as canopy shape and root architecture, may play a role in niche differentiation in polyclonal plantings. The complimentary canopy structure of different clones in mixed plantings may enhance photosynthetic capacity, leading to increased growth (blue arrows) and resource use (yellow arrows). Phenotypic plasticity may lead to exaggerated morphological differences, facilitating greater niche partitioning and resource use.

Previous studies have demonstrated that interspecific and intraspecific interactions between trees can significantly influence productivity, leading to notable changes such as enhanced photosynthetic capacity (Duan et al., 2014), alterations in root architecture (Richards et al., 2010), increased nitrogen uptake, and variations in leaf area index (LAI). A higher LAI often correlates with greater light interception and improved carbon assimilation, which can further enhance photosynthetic efficiency and contribute to overall productivity (Barigah et al., 1994). Several studies have shown that tree species diversity can increase productivity (Pretzsch, 2005; Erskine et al., 2006; Forrester et al., 2006; Kelty, 2006; Piotto, 2008; Richards et al., 2010). The plastic *Populus* spp. display high natural phenotypical variation related to its geographical distribution, and high intraspecific variability in traits (McKown et al., 2014). Because of the high level of phenotypic variability from *Populus* spp. (Bradshaw et al., 2000), polyclonal plantings of *P. deltoides* genotypes may be a reasonable analog for mixed species plantings (Bradshaw et al., 2000; Brunner et al., 2004). In contrast to monoclonal stands, individual trees with varying physiological (e.g., nitrogen-use efficiency) and morphological traits in polyclonal plantings can enhance soil characteristics by adjusting their fine root architecture. This adaptation improves nutrient uptake within their root zones, leading to increased soil nutrient utilization and overall productivity (Richards et al., 2010). Additionally, the greater productivity observed in *Populus* polyclonal plots may stem from reduced competition due to niche differentiation (**Figure 1**).

Changes in gene expression resulting from interactions between *P. deltoides* in polyclonal plantings could help target previously identified gene-biomarker associations or candidate genes related to specific mechanisms contributing to differences in polyclonal performance relative to monoclonals, such as enhanced growth and resource use efficiency for future testing in *P. deltoides* (Richards et al., 2010). Previous genome-wide association studies of *P. trichocarpa* have identified SNPs associated with key traits like height and volume gain (McKown et al., 2014; Fahrenkrog et al., 2017; Chhetri et al., 2019). Investigations into root morphology and transcriptomic reprogramming in *P. tremula × P. alba* revealed distinct changes in metabolic processes associated with nutrient acquisition (Wei et al., 2013). Similarly, research on *P. simonii* roots has highlighted shifts in growth allocation patterns between aboveground and belowground components (Zhang et al., 2018). Further studies have focused on leaf size and development, such as comparative transcriptomic analyses of *P. deltoides* and *P. simonii*, which identified candidate genes involved in molecular mechanisms driving leaf growth (Zhang et al., 2021). Another study analyzed the effects of gene expression in *Populus* clones grown in monoclonal and mixed stands with black locust (*Robinia pseudoacacia*), finding distinct differences in expression patterns between mixed and pure stands, potentially tied to resource partitioning and complementary growth strategies (Kuchma et al., 2020). However, analyzing pure clones, such as pure *P. deltoides* clones, simplifies the interpretation of results by minimizing confounding factors due to their genetic uniformity, enabling a clearer focus on environmental or treatment effects on gene expression. However, when planted in field settings, clones may exhibit phenotypic differences due to gene-environment interactions. Although their genetic makeup remains unchanged, environmental factors can influence gene expression, resulting in observable differences between the clones.

There is limited information on gene expression profiling of *P. deltoides* related to productivity and nitrogen mitigation in the Southeast United States (Richards et al., 2010; Luo et al., 2015; Han et al., 2020; Kuchma et al., 2020). This study aims to identify differentially expressed genes and biological pathways that may be associated with enhanced productivity, greater nitrate content in leaves, and expanded canopy coverage in polyclonal plantings. By analyzing and reporting the gene expression of two *P. deltoides* clonal varieties grown in monoclonal and polyclonal plots, this study contributes to the genomic understanding of *P. deltoides* plantations in the southeastern United States and uncovers genetic mechanisms driving productivity and resource efficiency.

## 2 Materials and methods

### 2.1 Study area and planting schemes

This study was nested in a larger field trial (*Populus* in the Southeast for Integrated Ecosystem Services (PoSIES) Department of Energy (DOE) DE-EE0009280). Dormant 38 cm unrooted cuttings of selected eastern cottonwood were obtained from Big River Cottonwood Nursery in Winnsboro, LA. Prior to planting and to provide initial protection against cottonwood leaf beetles, cuttings were soaked in water and Admire Pro^®^ systemic insecticide at a rate of 0.14 fluid oz. per 1 gallon (1.09mL/L), and 2.5oz flumioxazin + 32oz glyphosate tank mix was applied to the ground as preemergent weed control. Cuttings were planted on April 21, 2021. The larger study had a split plot design where the whole plot factor consists of inoculation with a mixture of endophytic bacteria obtained from Intrinsyx Bio in Sunnyvale, CA and no inoculation (i.e., control) and the split plot factor was twelve treatments of different varieties planted in monoclonal and polyclonal plots, three of which were used for this study. Treatments consisted of two polyclonal plots (of S7C8 and 110412), two monoclonal plots of clone S7C8 and two monoclonal plots of clone 110412. All clonal planting treatments consist of 30 tree plots planted in a 6 rows × 5 columns arrangement on a 1.8m × 1.8m spacing. The single row of trees around the perimeter of each plot served as a buffer and only the inner 12 trees (4 rows x 3 columns) served as trial trees. Cuttings were planted near riparian areas adjacent to agricultural fields to intercept surface runoff and shallow ground water from an agricultural field before it enters the stream.

**Figure 2:**
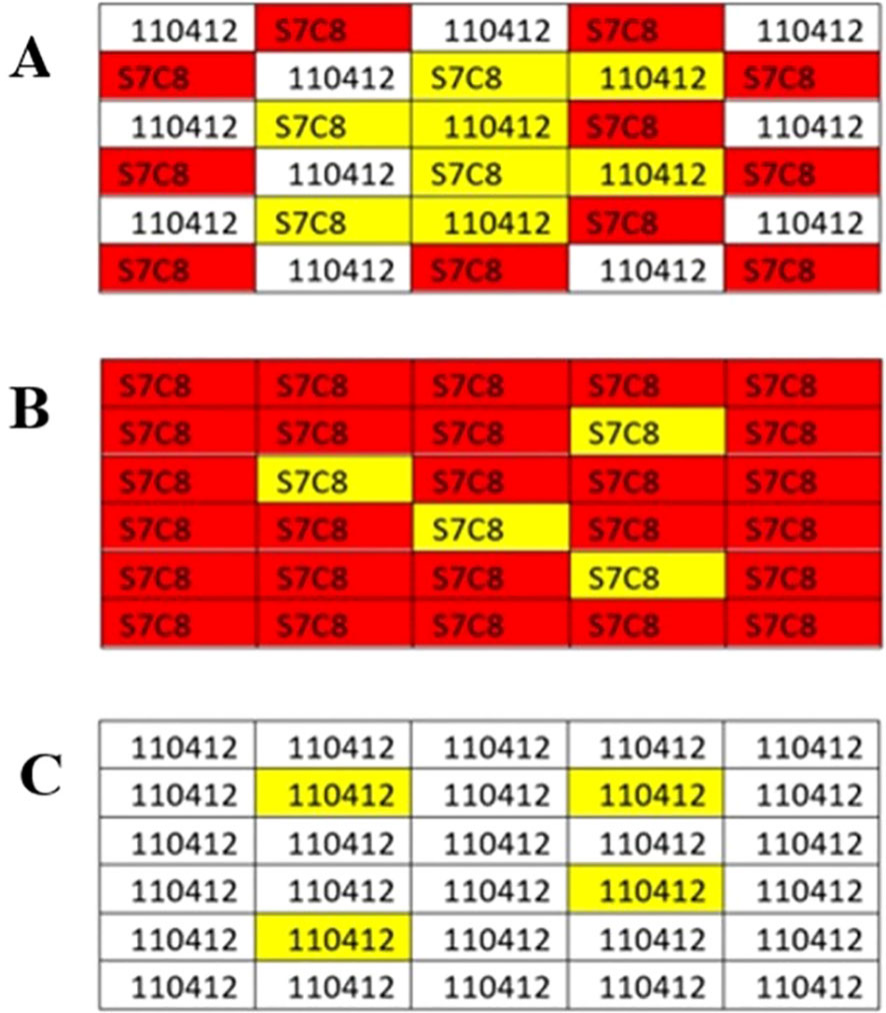
Polyclonal and Monoclonal planting design for one replicate for experimental analysis. **(A)** An example of eight individual biological replicates (highlighted in yellow) chosen for sampling analysis for polyclonal plantings. **(B, C)** An example of four individual biological replicates (highlighted) chosen for sampling analysis for monoclonal plantings. Leaf samples were chosen based on leaf quality and integrity.

Gene expression analysis focused on two *P. deltoides* (*P. deltoides* × *P. deltoides*) genotypes with contrasting nitrogen use characteristics based on data collected from previous field trials. Clone 110412 displayed lower nitrogen use efficiency and lower nitrogen percentage in leaf tissues compared to clone S7C8 (Renninger et al., 2022). Genotype “110412” originates from Bolivar County, MS in the Lower Mississippi Alluvial Valley and the genotype “S7C8” originated in Brazos County, Tx and was developed in the 1970s by the Texas Forest Service, Texas A&M, and the Western Gulf Tree Improvement Program (Jeffreys, 2005). These two genotypes were the only pure clones included in the broader trial. Samples and data were collected from monoclonal and polyclonal plots of 110412 and S7C8 in each of the main plots of a single complete replicate. Sequencing analysis was conducted on two replicate plots for each planting scheme for a total of four monoclonal plots (two of each clone) and two polyclonal plots (**Figure 2**).

### 2.2 Leaf material

At the end of July 2021 (peak of the growing season), leaves were collected from the six monoclonal and polyclonal plots for RNASeq and elemental combustion analysis. On July 28^th^, 2022, a hydraulic lift device was used to collect leaf samples from the terminal shoot. Between the fifth to twelfth leaf from the terminal, three fully expanded and undamaged leaf samples were cut from the tree for each individual biological replicate to ensure adequate material available for RNA extractions. Using a sterilized leaf cutter tool, leaf samples from four individual biological replicates (i.e. four trees) were taken from the planted monoclonal plots and leaf samples from eight individual biological replicates, four from each clone, were taken from planted polyclonal plots. All samples were collected within a three-hour window from 8 a.m. to 11 a.m. under consistent sunny conditions to minimize variations in gene expression related to temporal, and light changes between plots (Petrillo et al., 2015). At the beginning of the collection period, the average air temperature at an adjacent field site was 27.86°C at 8 a.m. and 32.74°C by 11 a.m. With a pair of sterilized scissors, one half of the three aggregated leaves were cut and placed in a 50ml labeled sample collection vial and flash frozen on dry ice and the other aggregated half was placed in a labeled Whirl-Pak^®^ write-on bag. Samples for elemental combustion analysis were placed in brown paper bags in an oven dehydrator at 60°C for 48 hours to remove excess moisture. Flash frozen samples for RNA extraction were placed into a freezer at -80°C until extractions occurred.

### 2.3 Determination of elemental composition in leaf tissue samples

Oven dehydrated leaf samples were removed from the oven and ground to a fine powder before placing them back in the dehydrator oven to dry overnight at 60°C. After drying, samples were placed in desiccators, allowing them to cool to room temperature for 1 hour. Between 2 to 4 mg of the homogenized leaf sample were weighed and used for subsequent elemental combustion analysis of total carbon and nitrogen (ECS 4010, Costech Analytical Technologies, Inc., Valencia, CA).

### 2.4 Groundwater collection

Shallow groundwater wells were installed upslope in the agricultural field side, and downslope in tree plots to assess agricultural nutrient runoff mitigation. Groundwater wells were established at half of the whole plot factor from the larger study. Control wells were established outside of plots on the agricultural field side to monitor changes in groundwater quality. Prior to sample collection, wells were manually evacuated and allowed to refill to ensure fresh groundwater collection. Water samples were placed on ice, returned to the laboratory, and stored at 4°C until processing. Nitrate (NO_3_^-^) concentrations were determined colorimetrically (AQ300 Discrete Analyzer, Seal Analytical) on samples that were filtered to remove particulates < 0.45μm. Groundwater samples collected biweekly during the growing season from June 3, 2021, through July 25, 2022. Multiple wells, including control wells were not sampled for all dates due to water depletion or water levels that dropped below a pump intake. Before performing statistical analysis, sampled dates containing no control wells and their associated planting schemes were removed from the dataset. Measurements excluded clonal information within respective planting schemes.

### 2.5 Biomass and leaf area index measurements

In December of 2021 all trees’ total height (with height poll) and diameter (with vernier calipers) at stem base were measured. A subset of trees were destructively sampled on September 22^nd^, 2021 throughout the larger study. In November and December of 2022, total height and diameter at breast (DBH) height (1.3m) of all trees were measured. Dry weights of sampled trees were utilized to develop allometric estimates of total aboveground biomass (including leaves) based on DBH and height. The allometric equation used to estimate aboveground biomass was:


ln(B)=a+b*ln(D)+c*ln(H)

where ln is the natural logarithm, B is the dry biomass in kg, D is the DBH in cm, H is the total height in meters, and a, b, and c are parameters. Measurements excluded clonal information within respective planting schemes.

Leaf area index (LAI) was measured nondestructively using an LAI 2200 (LiCOR Biosciences Inc., Lincoln, NE) on July 27^th^, 2022, one day before leaf collection for gene expression analysis. Measurements excluded clonal information within respective planting schemes.

### 2.6 Data analysis

Elemental combustion, groundwater and phenotypic measurements were analyzed in this study to coincide with gene expression sampling described below. For measurements lacking clone-specific information within their respective planting schemes, monoclonal plots were grouped by clone (clone S7C8 monoclonals, and clone 110412 monoclonals) and incorporated to evaluate overall clonal performance. To evaluate differences in nitrogen content (in mg) between planting schemes, a Mann-Whitney U test were utilized. For grouped clonal comparisons, we performed a Kruskal-Wallis Test. For comparisons within clones, we conducted and independent t-test for nitrogen content. In addition, an analysis of variance with Tukey’s procedure was conducted to assess differences in nitrate (NO_3_^-^) concentrations between planting schemes and among grouped clonal comparisons. Comparisons of total biomass and LAI between planting schemes were performed using an independent t-test and a Mann-Whitney U test. Grouped clonal comparisons were carried out with a Kruskal-Wallis Test followed by Dunn’s procedure for LAI. Clonal comparisons for total biomass were omitted due to low statistical power. All statistical tests were preformed in R version (v4.2.2; R Core Team, 2022).

### 2.7 Total RNA extraction, purification, and quality controls

Frozen leaf tissues (about 100 mg) were ground to a fine powder in liquid nitrogen using the sterilized mortar and pestle. Total RNA was extracted using the Qiagen RNeasy plant mini kit (Qiagen, Genmantown, MD, USA) following the manufacturer’s instructions. The quantity and quality of RNA was assessed with the Nanodrop™ One Spectrophotometer (Thermo Fisher Scientific, Waltham, MA, USA), and on a 1% (w/v) agarose gel. RNA samples were sent to Novogene (https://www.novogene.com/us-en/) for eukaryotic library creation and paired-end sequencing (PE150) on the Illumina NovaSeq 6000 (Illumina, San Diego, CA, USA). Novogene discarded paired-end reads for the following situations: when one read contains adapter contamination; when one read contains more than 10 percent of uncertain nucleotides; and when one read contains more than 50 percent low-quality nucleotides.

### 2.8 Gene expression analysis

The raw reads for each sample were mapped to the *Populus trichocarpa* (v4.1) transcriptome available from Phytozome (Goodstein et al., 2012). Salmon software (v1.8.0; Patro et al., 2017) was obtained from the GitHub repository (Patro, 2022) and run on the Linux kernel in the Ubuntu operating system (*Ubuntu* v20.04 *LTS*; Canonical Ltd., 2023, *Linux Kernel* v5.15; Torvalds, 2021), producing transcript-level expressions measured in transcripts per million (TPM). The transcript-level expressions were reduced to gene-level using R (v4.2.2; R Core Team, 2022) with the tximport package (v1.26.1; Soneson et al., 2016) and a custom-made transcript to gene map using the *P. trichocarpa* Phytozome annotations. Gene-level expressions were normalized according to the tximport manual and genes with low expression levels (fewer than 10 reads in more than 28 samples) were removed from further analysis using the R package edgeR (v3.40.2; Robinson et al., 2010). Differentially expressed genes were called using edgeR with the generalized linear model likelihood ratio test. Three models were fitted to the gene expression data: comparing polyclonal plots to baseline monoclonal plots (clone S7C8 + clone 110412 polyclonal vs. monoclonal) and comparing clones in their respective planting schemes (clone 110412 polyclonal vs. monoclonal, and clone S7C8 polyclonal vs. monoclonal) (**Table 1**). Transcripts with normalized log_2_ fold change > ± 1 and a BH adj. *p* <= 0.05 were considered differentially expressed genes (DEGs).

**Table 1:** Specified design matrices for differential expression analysis in *edgeR*.

Model Names	Design Matrix	Contrast	Output
1-Polyclonal vs. Monoclonal	~0+Planting+Clone+Plot	Polyclonal.vs. Monoclonal	Clone S7C8+Clone 110412 Polyclonal vs Monoclonal
2-Clone 110412 Polyclonal vs. Clone 110412 Monoclonal	~0+Planting: Clone+Plot	White.Poly.vs.Mono	Clone 110412 White Polyclonal vs White Monoclonal
3-Clone S7C8 Polyclonal vs. Clone S7C8 Monoclonal	~0+Planting: Clone+Plot	Red.Poly.vs.Mono	Clone S7C8 Red Polyclonal vs Red Monoclonal

The design matrix records which planting schemes were applied to each sample and defines how the experimental effects are parameterized in the linear models. Contrasts identify genes that are more highly expressed in polyclonal plantings. Each clone is designated with a specific color (Red: clone S7C8; White: clone 110412).

### 2.9 Gene set enrichment analysis

To interpret the DEG analysis output in a biological context, gene set enrichment analysis for both Gene Ontology (GO) and Kyoto Encyclopedia of Genes and Genomes (KEGG) were performed on each of the three comparison models using the fry method from the Limma R package (v3.54.1, Chen et al., 2015). GO gene sets were constructed using the GO terms in the *P. trichocarpa* v4.1 annotationGo terms with a *p* <= 0.01 were considered significant.

Since the Phytozome reference did not contain KEGG annotations, the Phytozome transcripts were mapped to the NCBI RefSeq *P. trichocarpa* (GCF_000002775.5) transcripts using the Basic Local Alignment Search Tool (BLAST+) (v2.13.0; Camacho and Madden, 2013), and vice versa. A transcript-to-gene database was created from ortholog determination using the Phytozome transcript IDs linked to the NCBI Entrez ID of the reciprocal best hit. The reciprocal best hit occurs when transcripts encoded by two genes from different genomes (*Populus trichocarpa* (v4.1) transcriptome and our sequenced *P. deltoides* transcriptome) identify each other as the best-scoring match in the opposite genome. The transcript-level estimates were reloaded using tximport and with this new database. The resulting gene-level expressions were normalized and filtered using the method described above. Pathways with a *p* or mixed *p* (disregarding gene direction) <= 0.01 were considered significant.

### 2.10 Primer design and RT-qPCR validation

Three transcripts, including *dehydration response element B1A* (*DREB1A*; Potri.015G136400), *exocyst subunit exo70 family protein H7* (*EXO70H7*; Potri.001G234600), and *oxidative stress 3 like 1* (*OS3*; LOC7491986) were selected and their expression levels were analyzed by the RT-qPCR assays to validate RNASeq results (see **Supplementary Material Table 3**). *DREB1A* was selected due to its significant differential expression, showing the highest expression in the polyclonal versus monoclonal planting model. *OS3* was chosen because it was consistently expressed across all three planting models, making it a reliable marker for comparison. *EXO70H7* was included because related exocyst components have been identified in *Populus* and are known to contribute to active growth by supporting cellular expansion (McKown et al., 2014).The gene-specific primers for each transcript were designed at the non-conserved regions based on the sequence alignment within each transcript and its homologous transcripts using BioEdit software (Hall, 1999). Each primer pair was selected to span at least one intron or locate at the exon-exon junction to ensure the amplification from the cDNA template.

One µg of DNase I-treated total RNA was used in reverse transcription reaction to synthesize the first-strand cDNA using Invitrogen SuperScript IV VILO Master Mix (Invitrogen/ThermoFisher Scientific, Waltham, MA, USA) according to the manufacturer’s instructions. The synthesized first-strand cDNA product was used as the template for RT-qPCR assays using ABI PowerUp SYBR Green Master Mix and ABI QuantStudio™ 5 Real-Time PCR System (Applied Biosystems/ThermoFisher Scientific, Waltham, MA, USA). The assays were conducted with three biological replicates per treatment group and three technical replicates per RNA (cDNA) sample. The qPCR program was set with an initial heating at 95°C for 2 min, followed by the denaturation at 95°C for 5 sec and the annealing/extension at 60°C for 25 sec for a total of 40 cycles. A dissociation curve (melting curve) analysis with a default setup was included after each real-time quantitative PCR run to validate the specific amplification of each transcript and the primer-dimer formation. The expression of poplar Ubiquitin gene (*UBQ; Potri.011G134200.1*) was used as a reference for normalizing the expression of each interested transcript in this study. The comparative Ct (2^-ΔΔCt^) method (Livak and Schmittgen, 2001) was used to analyze the expression differences.

## 3 Results

### 3.1 Elemental analysis

Nitrogen content in leaf tissue samples did not vary significantly between planting schemes, with an average of 0.06 ± 0.002 mg for both monoclonals and polyclonal plots (*p*=0.396). In grouped clonal comparisons, nitrogen content averaged 0.06 ± 0.003 mg for clone S7C8, 0.06 ± 0.001 mg for clone 110142, and 0.06 ± 0.002 mg for polyclonal plots, with no significant differences observed (*p*=0.748). For comparisons within clones, nitrogen content also showed no significant variation, averaging 0.06 ± 0.002 mg for monoclonals and 0.06 ± 0.001 mg for polyclonal plots in clone 110412 (*p*=0.460), and 0.06 ± 0.003 mg for both monoclonals and polyclonal plots in clone S7C8 (*p*=0.424).

### 3.2 Groundwater

Average nitrate concentrations showed significant differences between control wells and planting schemes (adj. *p*<0.05). However, multiple comparison tests did not reveal significant differences between the planting schemes, which had average nitrate concentrations of 1.59 ± 0.162 mg (N/L) for control wells, 1.21 ± 0.082 mg (N/L) for monoclonals, and 1.14 ± 0.119 mg (N/L) for polyclonal plots. In grouped clonal comparisons, monoclonal plots of clone S7C8 had significantly lower average nitrate concentrations compared to control wells (adj. *p*<0.05).

### 3.3 Survival, biomass and leaf area index

**Figure 3:**
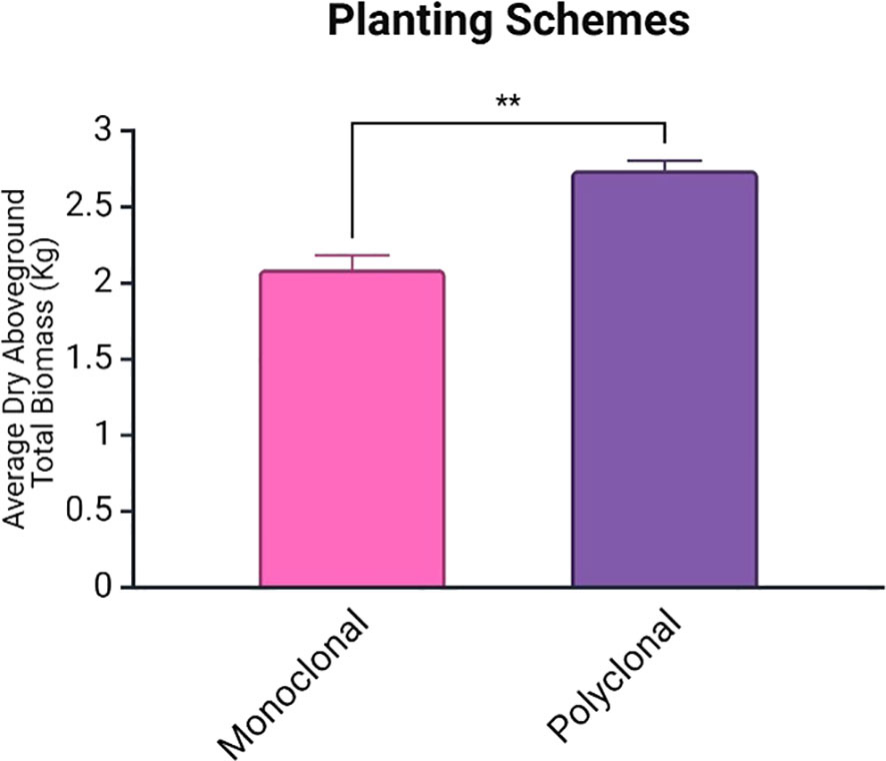
Average total biomass (including leaves) for planting schemes with standard error of mean bars. Average total biomass was significantly higher in polyclonal plots (*p* < 0.01). Biomass represents the total dry weight of all aboveground plant material (stems, leaves, branches). ** is statistically significant at 1% level.

At the end of the second growing season, there were 12 suriving trial trees in each of the polyclonal plots, 11 surviving trees in both S7C8 monoclonal plots, and 8 and 10 surviving trees in the 110412 monoclonal plots. Average total biomass was significantly higher in polyclonal plots (*p*<0.01: average of 2.09 ± 0.09 for monoclonals and 2.74 ± 0.06 for polyclonal plots) (**Figure 3**).

**Figure 4:**
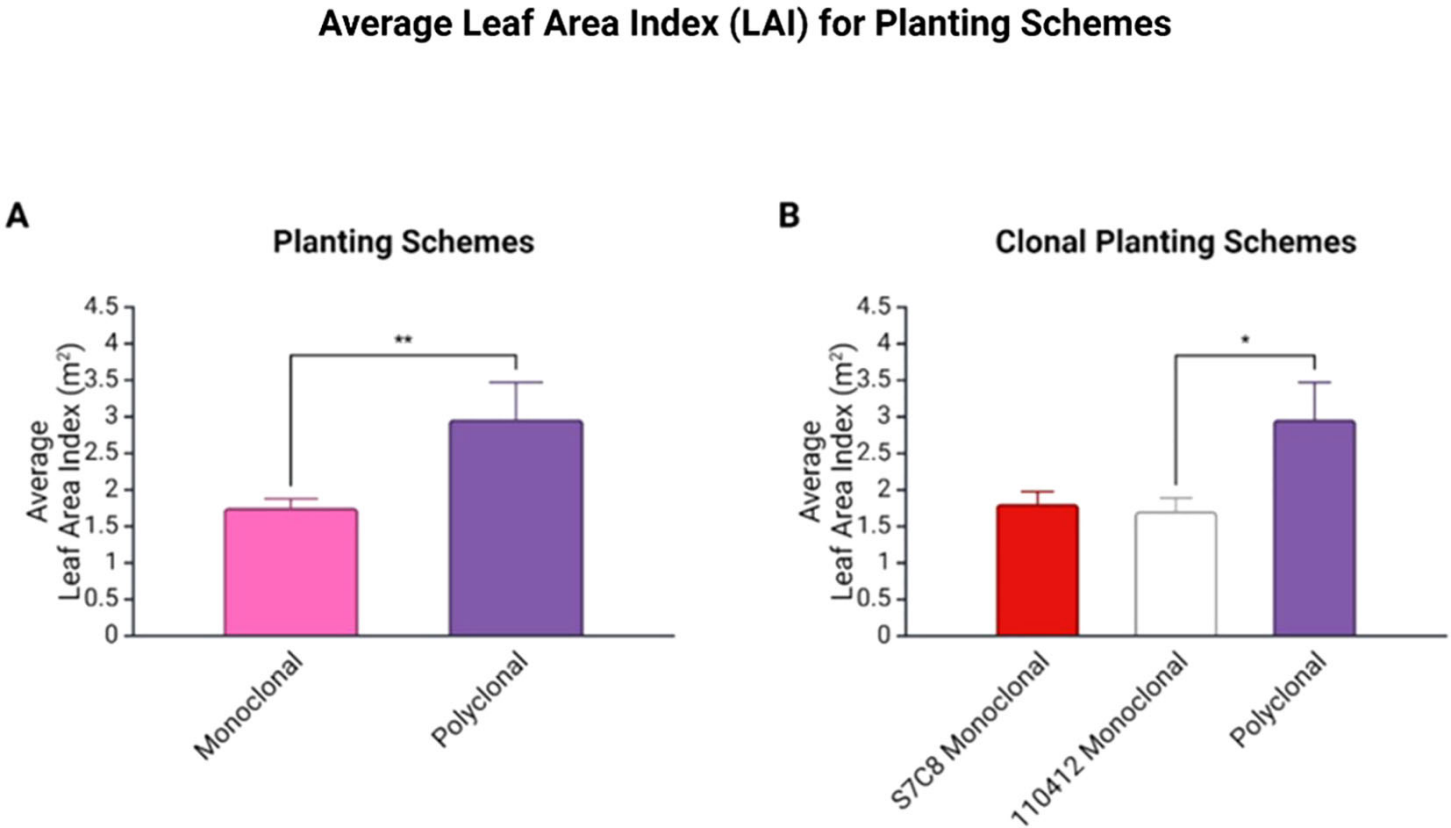
**(A)** Average Leaf Area Index (LAI) for planting schemes with standard error of mean (SEM). Average LAI was significantly higher in polyclonal plots (*p* < 0.01). **(B)** Average LAI for clonal planting schemes with SEM bars. Average LAI was significantly lower for clone 110412 compared to polyclonal plots (*p* < 0.05). * is statistically significant at 5% level. ** is statistically significant at 1% level.

Average LAI was significantly higher in polyclonal plots (*p*<0.01: average of 1.75 ± 0.124 m^2^ for monoclonals and 2.96 ± 0.057 m^2^ for polyclonal plots). Grouped clonal comparisons revealed significantly lower average LAI for clone 110412 compared to polyclonal plots (*p*<0.05) (**Figure 4**).

### 3.4 Differentially expressed genes

**Figure 5:**
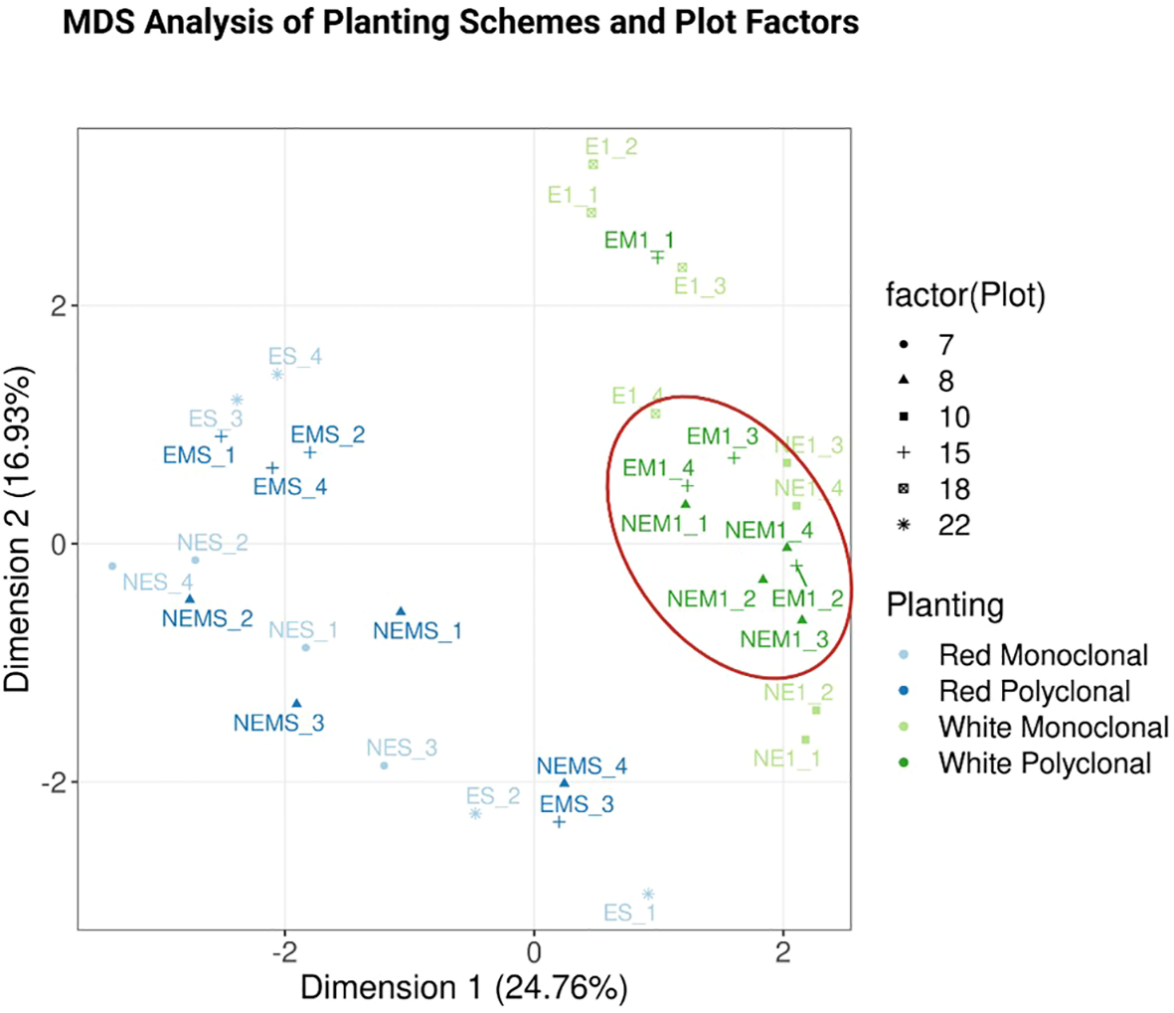
Multidimensional Scaling (MDS) Plot with clusters by planting scheme. This plot shows the relationship between samples, with each point representing a sample and the shape of each point indicating the six different plots from which samples were collected. Light blue and blue points correspond to clone S7C8 in monoclonal and polyclonal plantings, respectively, while light green and green points represent clone 110412 in monoclonal and polyclonal plantings. Monoclonal and polyclonal plantings for each clone cluster relatively close to each other. Notably, clone 110412 polyclonal plots are more distinctly clustered, as highlighted by the ellipse, compared to clone 110412 monoclonal plots. For clone 110412, the clustering may indicate clear and reliable separation, reflecting well-defined biological differences.

**Figure 6:**
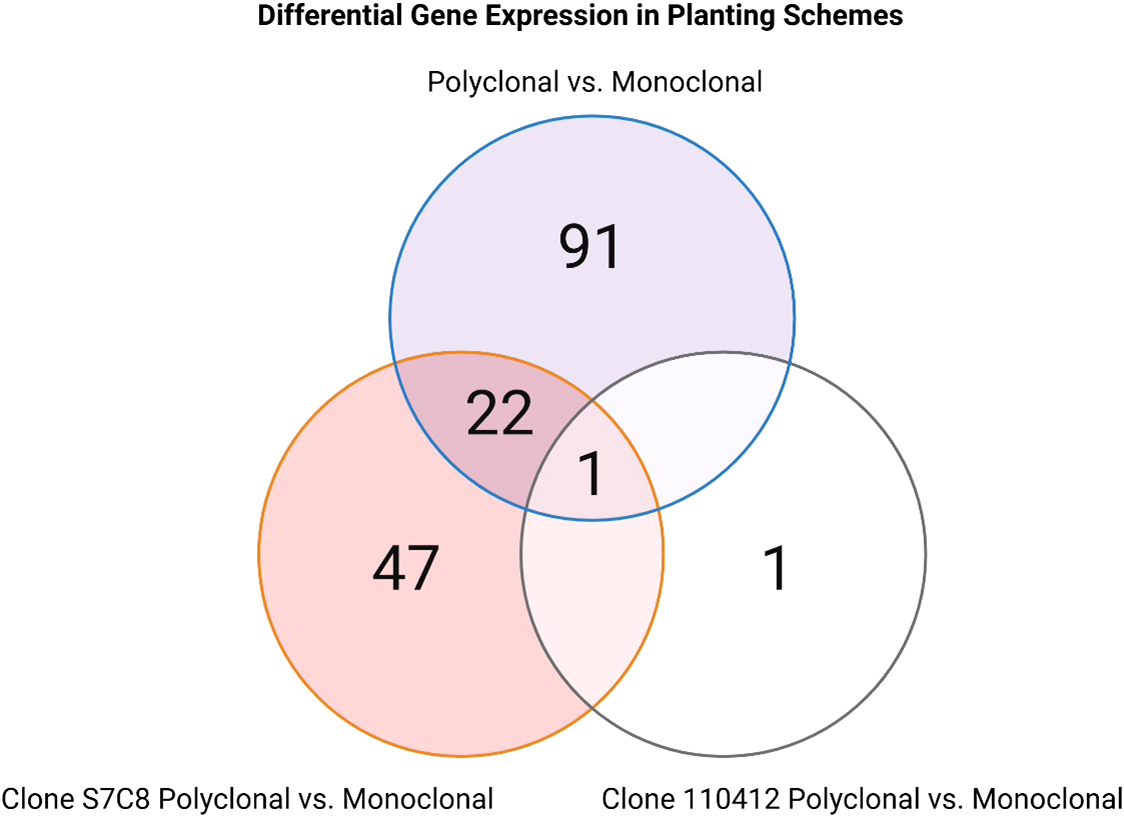
Venn diagram of differentially expressed genes across planting schemes. Each circle represents a different planting scheme, with numbers indicating the count of unique genes that are differentially expressed within that scheme. Overlapping regions show genes that are differentially expressed in multiple schemes, including one gene that is differentially expressed across all schemes.

**Figure 7:**
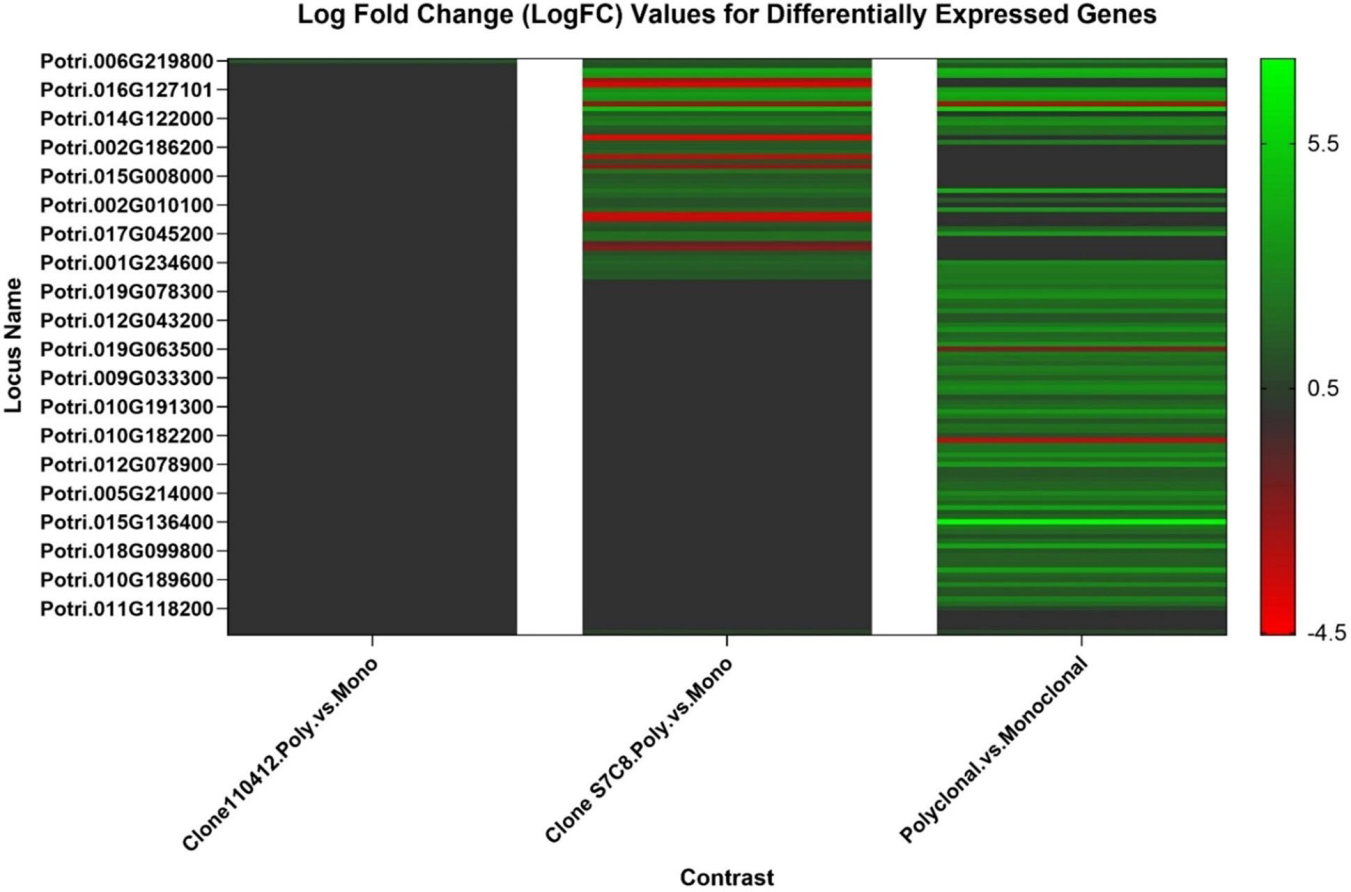
Fold change (from logarithmic scale base 2 (log_2_)) values for identified differentially expressed genes across all RNASeq models. Black indicates no identified differentially expressed gene for that model, green indicates upregulation and red indicates downregulation. Genes with parameters log_2_ fold change > ± 1 were included in the heatmap. See **Supplementary Material Table 1** for full differentially expressed genes list.

The samples were sequenced to an average depth of 46.44x reads (86,957,594 to 213,217,390 in ES_3 and NE1_4 respectively). The raw reads had a mapping rate averaging 87.63% across the entire transcriptome (71.99% in E1_4 to 90.74% in ES_1). The 52,400 transcripts from the reference genome corresponded to 34,627 genes at the gene-level expression. Of those, 26,783 genes passed the low expression filter. For the three models (i.e. comparing polyclonal vs. monoclonal, 110412 specific polyclonal vs monoclonal, and S7C8 specific polyclonal vs. monoclonal plantings), 91, one, and 47 genes were considered significantly (B.H. adjusted *p* <= 0.05) differentially expressed, respectively (**Figures 5**–**7**). The genes with the largest change in expression in the polycolonal compared to monoclonal plantings were *dehydration response element B1A* (Potri.015G136400; logFC: 7.24, B.H. adj. *p*: 0.025) and *prolyl oligopeptidase family protein* (Potri.002G014000; logFC: 5.77, B.H. adj. *p*:.007). Additional genes included *NDH-dependent cyclic electron flow 5* (Potri.019G034000; logFC:2.29, B.H. adj. *p*: 0.023), and *expansin-like A3* (Potri.009G141400; logFC: 2.57, B.H. adj. *p*<0.05). In clone 110412 polyclonal plantings, the only significant differentially expressed gene (Potri.06G219800; logFC:1.35, B.H. adj. *p*: 0.017) did not have functional annotations in Phytozome; however, the reciprical best hit orthlogue in the *P. trichocarpa* RefSeq dataset was *oxidative stress 3 like 1* (GeneID:7491986). The expression level of this gene was significantly different across all three treatments (clone S7C8 polyclonal vs clone S7C8 monoclonal, logFC: 1.33, B.H. adj. *p*: 0.003, polyclonal vs monoclonal logFC: 2.68, B.H. adj. *p* <0.001). Compared to clone S7C8 monoclonal plantings, the gene with the largest expression change in clone S7C8 polyclonal plantings was *prolyl oligopeptidase family protein* (Potri.002G014000; logFC: 4.92, B.H. adj. *p*: 0.005). Polyclonal vs. monoclonal and clone S7C8 polyclonal vs. clone S7C8 monoclonal expressed the same *exocyst subunit exo70 family protein H7* (polyclonal vs. monoclonal, Potri.001G234600, logFC: 3.07, B.H. adj. *p*: 0.003), with polyclonal plantings displaying upregulation for both models.

Gene ontology (GO) classified genes into one subcategory of cellular components for clone S7C8 polyclonal plantings. The GO term was identified as a proton-transporting v-type atpase, v1 domain (GO:0033, net direction: down, B.H. adj. *p*: 0.035). No additional models with significant B.H. adj. *p* values were identified for this study.

### 3.5 Pathway analysis of differentially expressed genes

The analysis revealed nine enriched KEGG pathways for clone S7C8 polyclonal vs. clone S7C8 monoclonal and no significant pathways for polyclonal vs. monoclonal and clone 110412 polyclonal vs. clone 110412 monoclonal at a 5% B.H. adj. p (**Table 2**). Multiple metabolic pathways were downregulated in clone S7C8 polyclonal plantings. The most significant downregulated pathway was arginine and proline metabolism (*p*<0.001, B.H. adj. *p* 0.026). Additionally, a mixed KEGG analysis was included to observe the magnitude of the expression change with a pathway, disregarding the direction of genes expressed. No pathways with significant B.H. adj. p values were identified for all three models for the mixed KEGG analysis.

**Table 2:** Enriched pathways in S7C8 polyclonal vs. monoclonal plantings.

PathwayID	NGenes	Direction	PValue	FDR	PValue.Mixed	FDR.Mixed	Description
pop00330	63	Down	0.00033	0.026	0.093	0.26	Arginine and proline metabolism
pop00960	36	Down	0.0005	0.026	0.011	0.19	Tropane, piperidine, and pyridine alkaloid biosynthesis
pop00514	16	Down	0.00056	0.026	0.046	0.25	Other types of O-glycan biosynthesis
pop00950	31	Down	0.001	0.033	0.009	0.19	Isoquinoline alkaloid biosynthesis
pop00400	54	Down	0.0012	0.033	0.0011	0.077	Phenylalanine, tyrosine, and tryptophan biosynthesis
pop00052	59	Down	0.0015	0.035	0.046	0.25	Galactose metabolism
pop00620	121	Down	0.0019	0.038	0.072	0.26	Pyruvate metabolism
pop00360	45	Down	0.0027	0.044	0.0054	0.19	Phenylalanine metabolism
pop00350	56	Down	0.0028	0.044	0.023	0.19	Tyrosine metabolism

Columns include: PathwayID (unique identifier for each pathway), NGenes (number of differentially expressed genes in each pathway), Direction (indicating upregulation or downregulation; all pathways shown are downregulated), PValue (P-value for statistical significance), FDR (false discovery rate to account for multiple testing), PValue.Mixed (P-value using a mixed model approach), FDR.Mixed (false discovery rate for the mixed model P-value), and Description (brief description of each pathway).

### 3.6 RT-qPCR analysis

**Figure 8:**
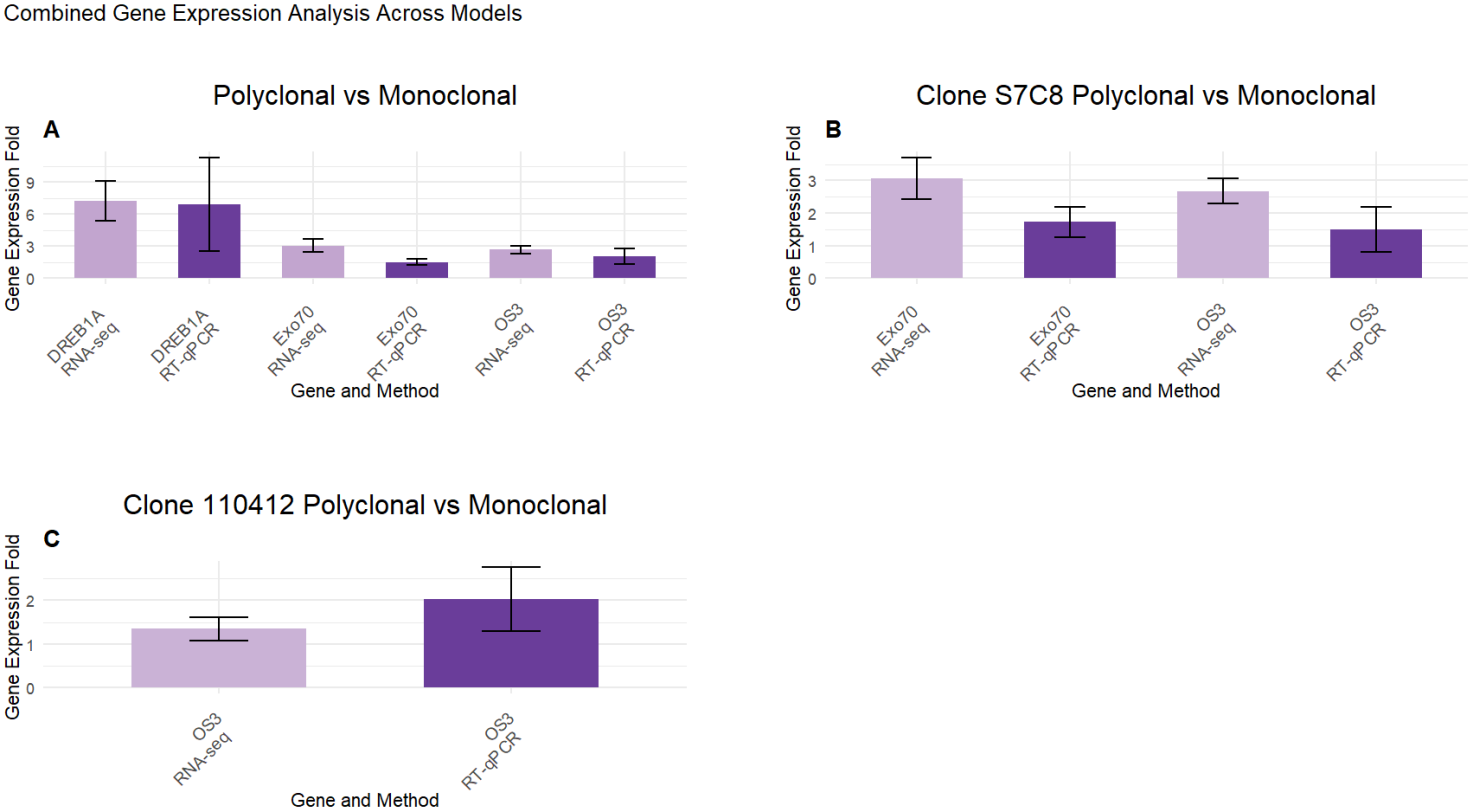
Combined gene expression analysis across models. Gene expression fold changes for **(A)** Polyclonal vs Monoclonal, **(B)** Clone S7C8 Polyclonal vs Monoclonal and **(C)** Clone 110412 Polyclonal vs Monoclonal models for RNASeq (LogFC) and RT-qPCR (relative fold change) analysis. The expression of three selected genes were compared against RNASeq data. Data are mean (SE) expressions from three biological replicates for each tested model. The Ubiquitin gene was used as an internal control for normalizing gene expression and fold change was calculated over monoclonal plots.

To confirm the reliability and validity of the RNASeq results within *Populus deltoides* planting schemes, we selected three genes, *DREB1A* with the largest fold change in our Polyclonal vs. Monoclonal model, *OS3* for its consistent expression across all models and *EXO70H7* as related exocyst components have been known to contribute to active growth in *Populus* (McKown et al., 2014). In comparisons between polyclonal and monoclonal groups, *DREB1A* showed an average fold increase in expression of 6.95 ± 4.38 in polyclonal plots and 1.75 ± 0.804 in monoclonals in leaf tissue samples, relative to our reference gene. For *EXO70H7* and *OS3* genes, the average fold increases were 1.51 ± 0.29 and 1.76 ± 0.44 in polyclonal plots, and 1.08 ± 0.18 and 1.034 ± 0.13 in monoclonals, respectively. In the S7C8 model, the *EXO70H7* gene exhibited an average fold increase in expression of 1.71 ± 0.51 in polyclonal plots and a fold decrease of 0.76 ± 0.16 in monoclonals. For the *OS3* gene, polyclonal plots in the S7C8 model showed an average fold increase of 1.49 ± 0.58, whereas monoclonals had an average increase of 1.18 ± 0.23. In the 110412 model, the *OS3* gene showed an average fold increase of 2.03 ± 0.74 in polyclonal plots and a and fold decrease of 0.88 ± 0.075 in monoclonals (see **Supplementary Material Table 2**). Overall, the selected genes showed a positive correlation with our RNASeq results (**Figure 8**). While the expression levels did not perfectly align, all selected genes demonstrated consistent expression trends, confirming the reliability of the transcriptome sequencing analysis.

## 4 Discussion

### 4.1 Pathway enrichment analysis highlights secondary metabolism and biosynthesis contributions to productivity and nitrogen use efficiency in *Populus* genotypes and planting schemes

Plants modulate secondary metabolism in response to stress, directly addressing stressors to maintain growth and productivity (Meraj et al., 2020). Amino acids such as arginine and proline play critical roles in this adaptive process, serving as precursors for secondary metabolites or as reservoirs of organic nitrogen (Baumberg and Klingel, 1993; Hildebrandt, 2018). The enhanced upregulation of secondary metabolic pathways, such as arginine and proline metabolism, observed in clone S7C8 monoclonal plantings, may reflect the clone’s sensitivity to abiotic stress. However, the association of stress responses with a high nitrogen use efficient clone could contribute positively to plant productivity by optimizing resource allocation under suboptimal conditions. As clone S7C8 is known for its high nitrogen use efficiency, the upregulation of arginine and proline metabolism may enhance growth, resilience and adaptation to stressors, particularly in monoclonal plots where resource competition or environmental pressures may be intensified (Richards et al., 2010). A similar scenario could explain the significant enrichment of the oxidative stress gene (*OS3*) across all models, with the highest expression levels observed in polyclonal plantings. This consistent upregulation in polyclonal plantings may indicate the role of an oxidative stress gene in mitigating cellular damage and maintaining homeostasis by managing reactive oxygen species (ROS) levels.

In addition, phenylalanine metabolism and the biosynthesis of phenylalanine, tyrosine, and tryptophan were significantly enriched in clone S7C8 monoclonal plantings. The phenylpropanoid pathway, which begins with phenylalanine deamination to produce p-coumaroyl CoA, is pivotal for synthesizing secondary metabolites that influence tree growth and productivity (Ma et al., 2018). Compounds derived from this pathway, such as phenolic glycosides, play a dual role in defense and stress adaptation while potentially impacting growth rates (Tsai et al., 2006). Phenolic glycosides, for instance, are more abundant in younger trees and have been shown to protect against pathogens and pests, contributing indirectly to productivity (Lindroth and Hwang, 1996; Boeckler et al., 2011). In clone S7C8, increased phenylalanine metabolism in monoclonal plantings may indicate enhanced adaptation to stress, but it could also reflect higher energy allocation to stress response at the cost of growth under certain conditions. Interestingly, polyclonal plantings demonstrated higher average aboveground biomass compared to monoclonal plots, suggesting that the presence of multiple clonal varieties alleviates stress impacts and enhances productivity. The benefits of polyclonal systems may stem from reduced intraspecific competition, greater niche complementarity, and more efficient resource use, particularly in stressful environments. For instance, reduced stress intensity in polyclonal plots likely allowed clone S7C8 to allocate more resources toward growth rather than stress responses, contributing to the observed biomass increases. This contrasts with monoclonal plantings, where heightened competition or reduced crown closure could exacerbate stress, as indicated by the upregulation of secondary metabolites, including alkaloids and phenolics. Secondary metabolites such as isoquinoline alkaloids, O-glycans, and tropane, piperidine, and pyridine alkaloids were also upregulated in clone S7C8 monoclonal plantings. While these compounds are not directly involved in primary growth processes, they play essential roles in defense and intraspecific interactions (Inayat et al., 2020). However, their upregulation in monoclonal plots may indicate intensified competition among individuals, potentially due to greater interactions with herbaceous vegetation or reduced crown closure. Such competitive dynamics could limit productivity in monoclonal systems, reinforcing the advantages of polyclonal plantings for biomass accumulation and stress mitigation.

### 4.2 Molecular mechanisms underlying productivity in *P. deltoides* polyclonal plantings

Although the expression levels from our RNASeq and RT-qPCR did not match, the results consistently showed expression of the oxidative stress gene (*OS3*) across all three models, with polyclonal plantings displaying the highest upregulation. Additionally, *DREB1A* displayed a dramatic 150-fold increase in polyclonal plantings. This consistency in pattern validates the reliability of our results (Mulozi et al., 2023), including for *EXO70H7*. *DREB1A* is known to play a critical role in stress and drought responses in *Arabidopsis* (Su et al., 2013), and previous studies have highlighted its significant upregulation under heat stress, accompanied by increased proline levels and decreased pyruvate levels (Ren et al., 2019). Oxidative stress, marked by excessive hydrogen peroxide (H_2_O_2_) production, is often triggered by light stress, high light intensities, and elevated temperatures (Inzé and Montagu, 1995; Jiang et al., 2017). Our results suggest that oxidative stress is occurring in the polyclonal plantings, with significant changes in gene expression indicating a potential stress signaling response and associated coping mechanisms. Interestingly, clone S7C8 in polyclonal plantings showed reduced proline and arginine metabolism, suggesting that this clone may be better equipped to handle stress.

*EXO70H7*, a member of the *EXO70* family, exhibited significant upregulation in both polyclonal versus monoclonal plantings and within the clone S7C8. Notably, the fold change in *EXO70H7* expression was higher in polyclonal plantings, as revealed by our polyclonal versus monoclonal model. *EXO70* subunits are broadly involved in processes critical for plant development and resource allocation, although the functional specificity of individual family members is still being explored (McKown et al., 2014). *EXO70H7* appears to have a specialized expression pattern, with activity in specific tissues such as the root maturation zone, root hairs, and leaf mesophyll (Pečenková et al., 2020). These tissues are vital for nutrient uptake, water absorption, and photosynthesis, respectively, underscoring the gene’s role in fundamental plant growth processes.

Additionally, *NDH-dependent cyclic electron flow 5* (*NDF5)*, also exhibited notable expression patterns in polyclonal plantings. This gene contributes to photosynthetic efficiency and stress tolerance by protecting the photosynthetic apparatus from damage (Ishida et al., 2009). Its activity enhances the stability and functionality of chloroplasts, complementing the role of *EXO70H7* in supporting photosynthetic processes. Similarly, *expansin-like A3* (*EXLA3)*, plays a critical role in cell wall loosening, a process vital for plant growth and development (Abuqamar et al., 2013). *EXLA3* also adjusts cell wall flexibility in response to environmental changes, further supporting the plant’s capacity to grow efficiently and adapt under varying conditions.

The mesophyll, as the primary site of photosynthesis, would directly benefit from enhanced exocyst-mediated vesicle trafficking facilitated by *EXO70H7*, alongside the protective and photosynthetic efficiency roles of *NDF5*. This synergy likely improves chloroplast function, stomatal regulation, and overall cellular efficiency, contributing to the increased LAI observed in clone S7C8. The activity of *EXLA3* in loosening cell walls would further promote larger or more flexible leaf development, which aligns with the observed increase in LAI. A higher LAI correlates with enhanced photosynthetic capacity, allowing plants to capture and utilize light resources more effectively, ultimately driving increased biomass production (Barigah et al., 1994).

In *Arabidopsis thaliana*, *EXO70H7* was found to be expressed in multiple cell types within the root meristem, indicating a diversification of function beyond conventional exocytosis (Li et al., 2010). This diversification may include specialized roles in root architecture and development, enhancing nutrient and water acquisition and indirectly supporting above-ground biomass accumulation. Similarly, in our study, clone S7C8 demonstrated elevated biomass production, which may be partly attributed to the upregulation of *EXO70H7*, *NDF5*, and *EXLA3*. This coordinated upregulation likely provides S7C8 with a competitive edge in terms of growth efficiency and adaptability, particularly in the diverse conditions of polyclonal plantings.

While these findings highlight the potential roles of *EXO70H7*, *NDF5*, and *EXLA3* in driving productivity, limitations in collecting and analyzing physiological data in field conditions pose challenges to fully understanding these mechanisms. Variability in environmental factors, such as soil composition, light availability, and water distribution, can introduce noise into physiological measurements. Additionally, biological variability among clones and logistical constraints, such as sample sizes may limit the resolution of observed trends. The time-sensitive nature of field measurements, such as midday gas exchange, further complicates data collection. Controlled environment experiments integrated with field trials would provide a robust framework for validating findings and reducing the influence of environmental variability.

### 4.3 Nitrate concentration and leaf area index for polyclonal plantings of *P. deltoides*

Our phenotypic and pathway enrichment analyses suggest that certain genes may significantly influence nitrogen metabolism in *Populus deltoides*, warranting further investigation. In *Populus*, leaf area, biomass production, and nitrogen levels are tightly interconnected, forming a foundation for plant growth and productivity (Cooke and Weih, 2005; Li et al., 2012). Differences in nitrogen-use efficiency among clones could have profound impacts on plant morphology, as demonstrated in previous studies showing distinct root system adaptations between slow-growing and fast-growing *Populus* species under varying nitrogen levels (Luo et al., 2013). Our findings reveal a reduced LAI in monoclonal plots at the peak of the growing season, which contrasts with the increased LAI observed in polyclonal plots. The elevated LAI in polyclonal plantings suggests a higher nitrogen uptake efficiency, potentially facilitated by more diverse root system structures or complementary resource acquisition strategies. This greater nitrogen absorption likely stems from enhanced utilization of groundwater and surface runoff, resulting in an increased leaf mass and higher total nitrogen content in polyclonal plots. Interestingly, despite the greater nitrogen uptake, the percentage of nitrogen content in leaf tissues remained similar between monoclonal and polyclonal plantings, indicating that polyclonal plots effectively distributed absorbed nitrogen across a larger leaf area, boosting canopy productivity.

These results align with our niche differentiation hypothesis, suggesting that the presence of multiple clonal varieties in polyclonal plantings enables more efficient resource partitioning and reduces interplant competition for nitrogen. Such partitioning likely enhances root exploration of soil layers and improves the capacity to capture nitrogen in polyclonal plots. This efficiency may lead to the formation of larger canopies with higher total leaf area, contributing to increased photosynthetic potential and biomass accumulation. Morphological changes, such as increased LAI, are critical indicators of productivity, as they directly influence light capture, gas exchange, and carbon assimilation in plant systems.

Although the LAI results highlight the advantages of polyclonal plantings, additional physiological data will provide a more comprehensive understanding of plant performance. Measurements of leaf gas exchange, including photosynthetic rates and stomatal conductance, will help determine whether increased LAI corresponds to greater photosynthetic activity. Moreover, evaluating water-use efficiency in leaf tissue samples will offer insights into how nitrogen metabolism interacts with water availability, particularly in diverse planting systems. Together, these data will clarify the mechanisms by which polyclonal plantings achieve superior growth and productivity, reinforcing the importance of variation among clones in optimizing resource use and ecological resilience.

### 4.4 Conclusion

These results suggest that variation among clonal varieties in polyclonal plantings enhances resource use efficiency and productivity, as demonstrated by higher overall productivity compared to the average of each monoclonal planting (overyielding), as well as surpassing the productivity of either monoclonal planting individually (transgressive overyielding). This underscores the role of variation among clonal varieties in facilitating complementary resource use and promoting growth under varying environmental conditions. However, the study faced limitations, such as challenges in obtaining accurate experimental validations due to the inherent environmental variability of field-based experiments and the extended time required for trees to mature enough to observe overyielding interactions. Experiments that collect individual physiological data across diverse planting schemes will provide a more accurate representation of the physiological responses of *P. deltoides* in these settings.

Several factors may explain the discrepancies between the RT-qPCR results and RNASeq data. First, the reduced number of biological replicates (three instead of four) and the use of only half of the experimental plots for RT-qPCR analysis (due to cost constraints and the need for pairwise comparisons) may have limited our ability to fully capture the gene expression changes identified by RNASeq. Additionally, incorporating multiple time points for gene expression analysis, especially when the trees are fully established and mature, could help align RNASeq and RT-qPCR results more closely and provide deeper genomic insights into the interactions within *P. deltoides* planting schemes.

While this study primarily focused on estimating aboveground biomass, polyclonal plantings may allocate more biomass belowground, highlighting the need for further analysis of belowground biomass and root transcriptomes to better understand total productivity. Investigating root system architecture, nutrient acquisition efficiency, and carbon allocation patterns could provide valuable insights into the mechanisms behind these findings. Temporal differences between sampled plots may have influenced gene expression results, despite efforts to minimize variability, suggesting that incorporating additional plots across time points could account for broader genotype-specific contributions to productivity. Future studies incorporating more clones could help identify key traits and genes that optimize resilience and growth across diverse planting schemes.

These results provide a foundation for identifying potential candidate genes and their associations with traits that could enhance productivity in future *Populus deltoides* planting schemes. In intermixed clones, gene expression is highly variable due to genetic differences, niche partitioning, and unique environmental interactions, making it difficult to identify universal genes critical to productivity. Instead, future research should prioritize genes differentially expressed based on genetic background, environmental factors, and inter-clonal dynamics, as these may offer more meaningful insights. While this study offers preliminary insights into clonal interactions and performance, the findings underscore the importance of incorporating additional -omics approaches, such as SNP calling from whole genome sequencing, to pinpoint genetic variations in candidate genes and identify genetic markers associated with specific productivity traits. Combining multiple -omics approaches would enable researchers to develop a comprehensive, systems-level understanding of how transcriptional changes influence downstream biological processes. To fully realize this potential, future trials validating candidate genes will be essential for advancing the development of *P. deltoides* clones with enhanced productivity and resilience, particularly in the context of climate change.

## Data Availability

The datasets presented in this study can be found in online repositories. The names of the repository/repositories and accession number(s) can be found in the article/**Supplementary Material**.
