# Supplementary material for: Comparative transcriptomic and phenotypic analysis of monoclonal and polyclonal Populus deltoides genotypes
Source: Front Plant Sci. 2025 Jan 23;15:1498535. doi: 10.3389/fpls.2024.1498535 (PMC11798960; doi:10.3389/fpls.2024.1498535)
Supplement: Supplementary file 3 [file Table1.docx]

**Supplementary Table 3.** List of primers selected for genetic expression in *Populus deltoides* leaf tissues

| Name | Locus ID | Primer | Amplicon Size (bp) |
| --- | --- | --- | --- |
| *dehydration response element B1A* | Potri.015G136400 | F: 5’-GTTGTCAGTTCTTGACGAGCAAGAATG-3’  R: 5’-CTTCACAAACCCACTTACCCGAATTCC-3’ | 233 |
| *oxidative stress 3 like 1* | Potri.006G219800 | F: 5’-GCAGTGGATCATAATAGTTGTAATAGCG-3’  R: 5’-CTTGTGAAAGACTTTGATTTGCCATTATAG-3’ | 294 |
| *exocyst subunit exo70 family protein H7* | Potri.001G234600 | F: 5’-CTTTCGGTAGCCAGAAAGCTTGTCC-3’  R: 5’-GACAGTGAGGAAGATGACGAAAGATGAC-3’ | 179 |
| *Ubiquitin Housekeeping Gene* | Potri.011G134200.1 | F: 5’-CGATAATGTGAAGGCCAAAATTCAG-3’  R: 5’-GGTCAGGGGGTATTCCTTCCTTGTC-3’ | 278 |
